# Using incentives to recruit physicians into behavioral trials: lessons learned from four studies

**DOI:** 10.1186/s13104-017-3101-z

**Published:** 2017-12-28

**Authors:** Deepika Mohan, Matthew R. Rosengart, Baruch Fischhoff, Derek C. Angus, David J. Wallace, Coreen Farris, Donald M. Yealy, Amber E. Barnato

**Affiliations:** 1 Department of Critical Care Medicine, University of Pittsburgh, 638 Scaife Hall, 3550 Terrace St, Pittsburgh, PA 15261 USA; 2 Department of Surgery, University of Pittsburgh, Pittsburgh, PA USA; 3 Department of Engineering and Public Policy, Carnegie Mellon University, Pittsburgh, PA USA; 4 RAND, Pittsburgh, USA; 5 Department of Emergency Medicine, University of Pittsburgh, Pittsburgh, PA USA; 6 The Dartmouth Institute, Geisel School of Medicine at Dartmouth, Lebanon, NH USA

**Keywords:** Recruitment, Response rates, Physicians, Questionnaires, Behavioral trials, Heuristics, Video games, Trauma, Guidelines, Physician adherence

## Abstract

**Objective:**

To describe lessons learned from the use of different strategies for recruiting physicians responsible for trauma triage, we summarize recruitment data from four behavioral trials run in the United States between 2010 and 2016.

**Results:**

We ran a series of behavioral trials with the primary objective of understanding the influence of heuristics on physician decision making in trauma triage. Three studies were observational; one tested an intervention. The trials used different methods of recruitment (in-person vs. email), timing of the honorarium (pre-paid vs. conditional on completion), type of honorarium [a $100 gift card (monetary reward) vs. an iPad mini 2 (material incentive)], and study tasks (a vignette-based questionnaire, virtual simulation, and intervention plus virtual simulation). We recruited 989 physicians, asking each to complete a questionnaire or virtual simulation online. Recruitment and response rates were 80% in the study where we approached physicians in person, used a pre-paid material incentive, and required that they complete both an intervention plus a virtual simulation. They were 56% when we recruited physicians via email, used a monetary incentive conditional on completion of the task, and required that they complete a vignette-based questionnaire.

*Trial registration* clinicaltrials.gov; NCT02857348

## Introduction

Understanding how physicians think is necessary to ensure that patients receive timely, safe, efficient, and effective care [1]. Behavioral trials allow insight into physician decision making. For example, they have revealed the influence of social networks on referral patterns, discretionary interventions on variation in cost, and social norms on treatment decisions for critically ill patients [2–5]. One major challenge to the successful completion of these studies is the recruitment of physician subjects [6, 7].

Reasons for reluctance to participate may include limited time, concerns about study validity, and reluctance to contribute to research that might reflect negatively on physicians’ own practice patterns [7–9]. Multiple studies have found that monetary incentives increase completion rates [7, 9, 10]. Much less is known about the effects of non-monetary material incentives.

In this paper, we report our experience recruiting and retaining physicians in four trials designed to understand how physicians make trauma triage decisions [11–14] We describe recruitment and retention rates in studies that used different: (a) modes of recruitment, (b) types of incentives, (c) timing of incentives, and (d) type of study tasks.

## Main text

### Materials and methods

#### Overview

We conducted four behavioral trials in the United States, between 2010 and 2016, to understand the influence of heuristics in trauma triage [11–14] Trauma triage involves a decision made under conditions of time-pressure and uncertainty, which has a well-accepted reference standard [15]. As such, it is a useful exemplar of a time-sensitive condition. These studies included: the development of a vignette-based instrument to analyze determinants of decision making (Study 1); [11] validation of the instrument by measuring retest reliability (Study 2a), known group performance (Study 2b), and external validity (Study 2c); [12] the development and validation of a virtual simulation (Study 3) [13], and a randomized trial evaluating a video game intervention for recalibrating heuristics (Study 4) [14].

We recruited physicians responsible for trauma triage, including physicians staffing emergency medicine departments and trauma surgeons. The study protocols were approved by the University of Pittsburgh Institutional Review Board, and informed consent was obtained before inclusion in the studies. Study 4, a clinical trial, was registered on clinicaltrials.gov (NCT 02857348).

We summarize key elements of each study in Table 1. Major differences among the four studies include: the method of recruitment, type of honorarium, timing of the honorarium, and nature of the study tasks. We describe these in detail, below.

**Table 1:** Summary of characteristics of studies

Study	Year	Description of study	Method of recruitment	Required task	Payment	Response and retention rate
1	2010	Development of a vignette-based instrument to measure physician performance	In person at a national meeting	Complete vignette-based instrument online at their convenience (1 h)	$100 gift card redeemable at ATM or in stores provided on enrollment and activated on completion of task	71% response rate; 62% completion rate
2	2011	Validation of a vignette-based instrument to measure physician performance: a) measurement of retest reliability, b) known groups validity, and c) external validity	a. Re-test reliability: email to participants of Study 1	Complete vignette-based instrument online at their convenience (1 h)	$100 Amazon gift card provided by email on completion of task	a. Re-test reliability: completion rate 64%
			b. Known groups validity: email to personal contacts with snowball recruiting			b. Known groups validity: completion rate 88%
			c. External validity: email to distribution list of healthcare organization in western Pennsylvania			c. External validity: completion rate 56%
3	2013	Development and validation of a virtual simulation to study physician decision making	In person at a national meeting	Complete virtual simulation online at their convenience (1 h)	$100 gift card redeemable at ATM or in stores provided on enrollment and activated on completion of task	79% response rate; 68% completion rate
4	2016	Randomized trial to test the efficacy of a video game intervention	In person at a national meeting	Complete intervention and then virtual simulation (minimum time-2 h)	Provided with iPad mini (approximate value $260) at the time of enrollment that they kept as their honorarium	88% response rate; 80% completion rate

#### Participants

In Studies 1, 3, and 4, we recruited national convenience samples of physicians working at non-trauma centers, while attending three different meetings of the American College of Emergency Physicians (2011–Las Vegas; 2013–Seattle; 2016–Las Vegas). Physicians were eligible if they practiced in non-trauma centers in the United States and made triage decisions for adult patients, regardless of their primary board certification. The cohort consisted primarily of emergency medicine physicians, but also included those with board certification in family practice, internal medicine, and general surgery. Physicians were ineligible if they practiced solely at Level I/II trauma centers, worked outside the United States, or managed only pediatric patients. In Study 2, we recruited with email letters: in Study 2a, we approached physicians who had participated in Study 1; in Study 2b, we approached a national convenience sample of trauma surgeons working at Level I/II trauma centers in the United States using personal contacts and snowball recruiting; in Study 2c, we approached physicians working at in the emergency department of UPMC non-trauma facilities in Pittsburgh, using a staff distribution list.

In each study, we established the sample size required to answer the study question by using Cohen’s method of estimating power for behavioral trials, and assuming a 60 (Studies 1, 2, 3) to 70% (Study 4) completion rate.

#### Recruitment strategies

For Studies 1, 3, and 4, we hired a booth in the Exhibition Hall of the meeting. When physicians approached us, we described the study procedure, noting both our National Institutes of Health (NIH) support and the scientific value of the research. Finally, we mentioned the honorarium for participation. For Studies 1 and 3, we provided a $100 University of Pittsburgh WePay MasterCard gift card at the time of enrollment, and activated the card once the participants completed the task. For Study 4, we provided an iPad mini 2 (approximate value $260) at the time of enrollment, which physicians could keep at the conclusion of the trial.

For Study 2, we recruited physicians through email messages describing the study, also noting the $100 honorarium. We sent participants an Amazon gift card by email after they had completed the task.

#### Retention strategies

The principal investigator (PI) sent personalized email reminders to physicians at 1 week intervals for 1 month after enrollment. These messages reminded participants about study procedures and emphasized the value of their participation.

#### Study procedures

In Studies 1–3, we asked physicians to log into a secure website, respond to a demographic survey, and complete an instrument designed to assess their performance in triage decisions. That instrument was either a vignette-based questionnaire or a virtual simulation. We scored responses against the reference standard set by the American College of Surgeons. In Study 2, we additionally compared within-subject responses to the questionnaire at two separate time points (Study 2a: re-test reliability), compared the responses of trauma surgeons and emergency physicians enrolled in the study (Study 2b: construct validity), and compared responses to decisions these same physicians had made in practice (Study 2c: external validity). Completion of the study procedures took approximately 1 h.

In Study 4, we randomized physicians to one of two interventions (video game or educational program). We asked participants to use their intervention for 1 h, and then login to a secure website to complete the virtual simulation developed in Study 3. We scored responses, and then compared performance between groups and determine the effect of the video game on performance. Completion of the study procedures took a minimum of 2 h.

#### Statistical analyses

We summarize recruitment and retention rates among studies using descriptive statistics. We avoided further statistical analysis since we did not have a priori hypotheses to test. We used Stata 13.0 (Statacorp, TX, USA) for data management and analysis.

### Results

#### Participant characteristics

We recruited 280 physicians for Study 1, 132 physicians for Study 2, 209 physicians from Study 3, and 368 physicians for Study 4. As shown in Table 2, physicians who completed the studies had a mean age of 41 years (SD 9.5) and approximately 10 years (SD 9.2) of experience. They were mostly male (76%), white (75%), and trained in emergency medicine (93%). Most had received certification in Advanced Trauma Life Support (ATLS) (76%), and worked exclusively at non-trauma centers (83%).

**Table 2:** Participant characteristics presented with number of participants (percentage per study [%]) and means (standard deviation [SD])

Variable	Overall (N = 674)	Study 1 development of vignette-based questionnaire (n = 168)	Study 2 validation of vignette-based questionnaire			Study 3 development and validation of a virtual simulation (n = 142)	Study 4 testing the efficacy of a video game to reduce diagnostic error among physicians (n = 295)
			a Retest reliability (n = 32)	b Known groups (n = 28)	c External validity (n = 28)		
Age, mean (SD)	41 (9.5)	42 (9.6)	43 (8.7)	44 (6.9)	47 (8.8)	43 (10.7)	40 (8.9)
Male, n (%)	511 (76)	141 (84)	26 (81)	21 (75)	19 (68)	112 (79)	192 (65)
Race, n (%)							
White	503 (75)	127 (76)	27 (84)	20 (71)	23 (82)	105 (74)	201 (68)
Black	21 (3)	8 (5)	0 (0)	0 (0)		3 (2)	10 (3)
Asian	95 (14)	20 (12)	4 (13)	3 (11)	5 (19)	18 (13)	50 (17)
Latino	38 (6)	9 (5)	0 (0)	0 (0)		6 (4)	23 (8)
Native American or Pacific Islander	10 (1)	3 (2)	0 (0)	0 (0)		2 (1)	5 (2)
Other	21 (3)	1 (1)	1 (3)	2 (7)		8 (6)	8 (3)
Primary specialty							
Emergency Medicine	625 (93)	157 (93)	30 (94)	–	20 (71)	135 (95)	283 (96)
Family Practice/Internal Medicine	36 (5)	10 (5)	1 (3)	–	8 (29)	7 (5)	10 (4)
Trauma Surgery	28 (4)	–	–	28 (100)	–	–	–
Other	5 (1)	1 (1)	1 (3)	–	–	–	3 (1)
Years experience, mean (SD)	10.1 (9.2)	11.8 (9.2)	12 (8.9)	11.3 (7)	16 (9.8)	11.1 (10.6)	8.4 (8.5)
ATLS certified, n (%)	512 (76)	125 (74)	20 (63)	28 (100)	19 (68)	113 (80)	207 (70)
Physician also works at a Level I/II trauma center, n (%)	112 (17)	14 (8)	6 (18)	28 (100)	0 (0)	28 (20)	36 (12)

#### Participant recruitment

**Fig. 1:**
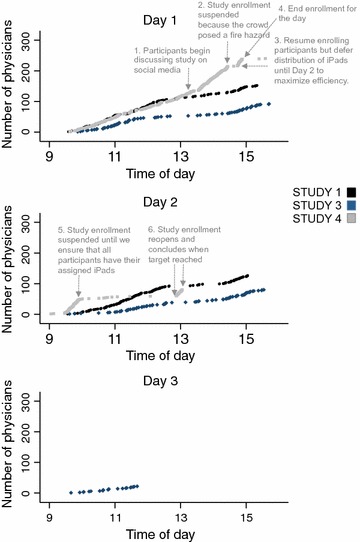
Rates of recruitment for Studies 1 (black), 3 (navy), and 4 (grey), where subjects approached in-person at a national meeting of the American College of Emergency Physicians

Figure 1 shows recruitment rates, over time, of the three studies that used an in-person strategy. Over the entire recruitment period, that rate ranged from 15 participants/h (Study 3) to 39/h (Study 4). In Studies 1 and 3, accrual occurred slowly. Two members of the study team stopped conference attendees who passed by the booth to screen for interest and eligibility. For Study 4, participants independently exchanged social media messages about the study after approximately 4 h, which increased our recruitment rates from 35/h (hours 2–4) to 61/h (hours 5–6).

Qualitatively, we made several observations during the recruitment process for Study 4 that suggested that the material incentive we provided (an iPad) assumed value disproportionate to its cost. First, physicians demonstrated a willingness to wait in lines of 50–60 people to undergo screening procedures. Second, they retained interest in participating despite external barriers to enrollment. At one point, we halted recruitment at the request of conference organizers, since the size of the crowd around our booth violated fire safety regulations. Physicians returned multiple times to determine whether and when we would re-open enrollment. Third, physicians expressed anger and frustration if they did not meet eligibility criteria, describing our explanation as “unfair.”

#### Participant retention

As shown in Table 1, completion rates were 56% in Study 2c, where we used email to recruit physicians with whom we had no prior relationship, required that participants complete a vignette-based questionnaire, and provided the honorarium at the conclusion of the task. Completion rates were 80% in Study 4, where we recruited physicians in person, required that participants complete an intervention and virtual simulation, provided the honorarium at the time of enrollment, and used a material, rather than a monetary incentive.

### Discussion

Physicians represent a particularly challenging group to recruit into behavioral trials, given their limited time and frequent skepticism about the value of such research. Rates of participation typically range from 35 to 54%. We learned that approaching physicians at a national conference allowed us to maximize the efficiency of recruitment, providing access to a large (albeit not necessarily representative) sample of physicians. Several systematic reviews describe the importance of variables such as such as contact with participants, the appeal of the study tasks, and incentives on recruitment and retention of subjects [10]. Our results validate these observations. However, in contrast to prior studies, we also learned that material incentives may work as well as monetary ones to encourage participation.

Prior efforts to use material incentives to increase response rates have had mixed success [10, 16]. Token non-monetary incentives (e.g. informational brochures, pencils) typically prove ineffective [17]. More substantial inducements, such as the opportunity to enter a lottery with a large payout, have sometimes, but not reliably, increased response rates [16, 18]. In contrast, our results suggest that certain non-monetary material incentives can encourage participation in a behavioral trial. Our choice of the particular incentive reflected practical considerations: pre-loading interventions on iPads facilitated completion of study tasks. We worried that many physicians would not be strongly motivated by this offer. Unexpectedly, we found that physicians found the honorarium extremely attractive, as demonstrated by our qualitative observations of subject behavior during the enrollment process of Study 4.

In this secondary analysis of trial enrollment data, we cannot disentangle the incremental effect of the different elements of the incentive. We speculate that a material incentive provided at the time of enrollment may have influenced behavior in three ways. First, we reduced the transactional costs involved in purchasing an iPad. In other words, physicians in our study may have valued an (additional) iPad, but were not willing to purchase it themselves when faced with the opportunity costs of spending fungible cash or time shopping. Second, it made the utility of the honorarium transparent for a population whose economic status might otherwise lead them to dismiss the value of a financial incentive. Finally, it expressed trust in participants to complete the task. Distinguishing the relative roles of these effects would require systematic experimental manipulation.

Behavioral research must distribute limited resources across task design, data collection, and data analysis. Those resources include investigators’ time and energy. Experiences like those summarized here provide inputs to the complex calculus of how to spend those resources. Our results suggest the potential value of providing attractive material rewards when attempting to recruit physicians into behavioral trials. However, they should be interpreted with caution given the homogeneity of our population and the descriptive nature of our observations.

## Limitations

The generalizability of these observations is limited by the type of decision we studied and the tasks involved. In addition, we used a wage payment model to set the size of our incentive and to limit the potential for undue inducement. In contrast, most other studies that use monetary incentives implicitly use either a free market or an appreciation model of reimbursement [19, 20]. We do not know how many physicians would have responded in the absence of any incentive, or to a payment set using a different model of reimbursement. However, we speculate that use of an incentive allows for the recruitment of a more generalizable population than reliance on altruism alone. Finally, our recruitment strategy, tailored to address our research question, resulted in the selection of a homogenous population. Some evidence exists that physician characteristics can influence response rates to questionnaires (e.g. internists typically have a higher response rate than general surgeons) [21]. Therefore, we cannot speculate on the generalizability of our results to other groups or types of physicians.

## Abbreviations

NIH: National Institutes of Health
PI: principal investigator
ATLS: Advanced Trauma Life Support
